# Characterization of *Aspergillus tamarii* Strains From Human Keratomycoses: Molecular Identification, Antifungal Susceptibility Patterns and Cyclopiazonic Acid Producing Abilities

**DOI:** 10.3389/fmicb.2019.02249

**Published:** 2019-10-09

**Authors:** Mónika Homa, Palanisamy Manikandan, András Szekeres, Noémi Kiss, Sándor Kocsubé, László Kredics, Bader Alshehri, Abdul Aziz Bin Dukhyil, Rajaraman Revathi, Venkatapathy Narendran, Csaba Vágvölgyi, Coimbatore Subramanian Shobana, Tamás Papp

**Affiliations:** ^1^MTA-SZTE “Lendület” Fungal Pathogenicity Mechanisms Research Group, Szeged, Hungary; ^2^Department of Microbiology, Faculty of Science and Informatics, University of Szeged, Szeged, Hungary; ^3^Department of Medical Laboratory Sciences, College of Applied Medical Sciences, Majmaah University, Al Majma’ah, Saudi Arabia; ^4^Greenlink Analytical and Research Laboratory (India) Private Limited, Coimbatore, India; ^5^Aravind Eye Hospital and Postgraduate Institute of Ophthalmology, Coimbatore, India; ^6^Department of Microbiology, PSG College of Arts & Science, Coimbatore, India

**Keywords:** *Aspergillus tamarii*, keratitis, antifungal drug susceptibilities, drug interactions, molecular identification, calmodulin, cyclopiazonic acid (CPA), LC-MS/MS

## Abstract

*Aspergillus tamarii* appears to be an emerging aetiological agent of human keratomycoses in South India. The investigated strains were isolated from six suspected fungal keratitis patients attending a tertiary care eye hospital in Coimbatore (Tamil Nadu, India), and were initially identified by the microscopic examinations of the scrapings and the cultures. Our data suggest that *A. tamarii* could be easily overlooked when identification is carried out based on morphological characteristics alone, while the sequence analysis of the calmodulin gene can be used successfully to recognize this species accurately. According to the collected clinical data, ocular trauma is a common risk factor for the infection that gradually developed from mild to severe ulcers and could be healed with an appropriate combined antifungal therapy. Antifungal susceptibility testing revealed that *A. tamarii* strains are susceptible to the most commonly used topical or systemic antifungal agents (i.e., econazole, itraconazole and ketoconazole) except for natamycin. Moreover, natamycin proved to be similarly less effective than the azoles against *A. tamarii* in our drug interaction tests, as the predominance of indifferent interactions was revealed between natamycin and econazole and between natamycin and itraconazole as well. Four and five isolates of *A. tamarii* were confirmed to produce cyclopiazonic acid (CPA) in RPMI-1640 – which is designed to mimic the composition of human extracellular fluids – and in yeast extract sucrose (YES) medium, respectively, which is a widely used culture medium for testing mycotoxin production. Although a ten times lower mycelial biomass was recorded in RPMI-1640 than in YES medium, the toxin contents of the samples were of the same order of magnitude in both types of media. There might be a relationship between the outcome of infections and the toxigenic properties of the infecting fungal strains. However, this remains to be investigated in the future.

## Introduction

Keratitis due to yeasts and filamentous fungi is a serious health issue in tropical and subtropical countries. In South India, it is among the leading causes of ocular morbidity and corneal blindness (Bharathi et al., 2003; Srinivasan, 2004), that could be avoidable with a timely diagnosis and a subsequent appropriate antifungal therapy (Gupta et al., 2013). Fungal keratitis typically affects middle-aged men having agriculture-related or other outdoor occupations in developing countries. While trauma has been the key predisposing factor, diabetes mellitus, contact lens wear, corticosteroid therapy, a prior eye surgery or eye disease are also among the frequently reported other risk factors for the infection (Bharathi et al., 2007; Thomas, 2009; Thomas and Kaliamurthy, 2013; Ranjini and Waddepally, 2016). Among filamentous fungal aetiologies, the genera *Fusarium* (31–52.5%) and *Aspergillus* (11–41%) are the main pathogens (Chowdhary and Singh, 2005; Ranjini and Waddepally, 2016; Manikandan et al., 2019). The spectrum of *Aspergillus* species identified from keratitis cases is relatively wide. While *A. flavus* is the most common cause of *Aspergillus* keratitis, *A. fumigatus*, *A. terreus* (Kredics et al., 2015), *A. brasiliensis* (Manikandan et al., 2010), *A. nomius* (Manikandan et al., 2009), *A. pseudotamarii* (Baranyi et al., 2013), *A. tamarii* (Kredics et al., 2007; Manikandan et al., 2013; Ghosh et al., 2016; Cuadros et al., 2018; Al-Hatmi et al., 2019; Manikandan et al., 2019), and *A. tubingensis* (Kredics et al., 2009) were also reported sporadically from human keratomycoses.

*Aspergillus tamarii* is a member of the *Aspergillus* section *Flavi*. It is widely used in the food and fermentation industries (Varga et al., 2011). At the same time, it has been more commonly recognized from human infections in the last 15 years. In addition to keratitis, this species has been reported from an eyelid infection (Degos et al., 1970), onychomycosis (Kristensen et al., 2005), cutaneous infections (Sharma et al., 2013; Kimura et al., 2018), a wound infection (Aries et al., 2018), an invasive nasosinusal aspergillosis (Paludetti et al., 1992), a nasal polyposis (Kamble, 2015) and respiratory tract samples (Tam et al., 2014; Castro et al., 2019). It is a non-aflatoxigenic species, although, its representatives are known for their cyclopiazonic acid (CPA), fumigaclavin and kojic acid producing ability (Varga et al., 2011).

Because of the small number of cases where molecular identification, antifungal susceptibilities and clinical data are all provided at the same time, clinical breakpoints and even epidemiological cutoff values are not available for this species. In order to determine the clinical relevance of identifying *A. tamarii* at the species level, studies like the present work are necessary. Here, we report and characterize six *A. tamarii* isolates from keratitis, and also provide a review of the relating English-language literature. Our specific goals were to compare and discuss the reliability of morphological and molecular identification methods in case of *A. tamarii*, to determine the antifungal susceptibility profiles of the isolates, to study the *in vitro* efficacy of clinically relevant dual drug combinations against the isolates, to examine the relationship between the *in vitro* antifungal susceptibility and the outcome of therapies, to determine the CPA producing ability of the strains, and finally, to compare our observations to the relevant literature of the field.

## Materials and Methods

### Molecular Identification

Six isolates of *Aspergillus* spp., obtained after processing the corneal scrapings from the suspected fungal keratitis patients attending the Cornea Services at Aravind Eye Hospital and Postgraduate Institute of Ophthalmology (Coimbatore, Tamil Nadu, India) during 2005 and 2007 and identified based on the initial morphological examinations (i.e., KOH wet mount of corneal scrapings, growth on 5% sheep blood agar, chocolate agar and potato dextrose agar, and micromorphology of the cultures after lactophenol cotton blue staining), were included in the study. For the species-level identification using molecular methods, the internal transcribed spacer (ITS) region and a part of the calmodulin gene were amplified using the ITS1/ITS4 and cmd5/cmd6 primer pairs, respectively, as described earlier (White et al., 1990; Hong et al., 2006). The sequences were determined at LGC Genomics GmbH (Berlin, Germany) and were deposited in the GenBank database under the accession numbers listed in Table 1. BLAST searches were conducted against the GenBank database at the website of the National Center for Biotechnology Information^1^ (Altschul et al., 1990).

**TABLE 1:** Results of the identification of *Aspergillus* spp. from keratitis.

**Case**	**Strain number**	**ITS**		**Calmodulin**		**Species based on**		
		**GenBank accession number**	**Length**	**GenBank accession number**	**Length**	**Morphology**	**ITS**	**Calmodulin**
1	SZMC 2393	MG907190	522	MG912921	520	*A. flavus*	*A. tamarii*	*A. tamarii*
2	SZMC 2404	MG907191	521	MG912922	516	*A. flavus*	*A. tamarii* or *A. nomius*	*A. tamarii*
3	SZMC 2428	MG907192	521	MG912923	516	*Aspergillus* sp.	*A. tamarii*	*A. tamarii*
4	SZMC 2434	MG907193	530	MG912924	516	*A. flavus*	*A. tamarii*	*A. tamarii*
5	SZMC 2438	MG907194	532	MG912925	516	*A. flavus*	*A. tamarii*	*A. tamarii*
6	SZMC 2439	MG907195	535	MG912926	516	*A. fumigatus*	*A. tamarii*	*A. tamarii*

*ITS, internal transcribed spacer; SZMC, Szeged Microbiological Collection, University of Szeged, Szeged, Hungary.*

All the six isolates have been deposited at the Szeged Microbiology Collection (SZMC, Szeged, Hungary^2^) under the accession numbers of SZMC 2393, SZMC 2404, SZMC 2428, SZMC 2434, SZMC 2438, and SZMC 2439.

### Antifungal Susceptibility Testing

Determination of antifungal susceptibility profiles of the six isolates was performed according to the guidelines of the CLSI M38-A2 broth microdilution method (Clinical and Laboratory Standards Institute, 2008). Eight clinically relevant antifungal agents, such as amphotericin B, natamycin, clotrimazole, econazole, fluconazole, itraconazole, ketoconazole and terbinafine (Sigma-Aldrich, United States) were included in the study. The final concentration range of amphotericin B, clotrimazole, econazole, fluconazole, ketoconazole and natamycin was 0.125–64 μg/ml, while in case of itraconazole and terbinafine, the tested concentrations ranged between 0.002 and 1 μg/ml. Microdilution test plates were evaluated after 48 h of incubation at 35°C. All plates were read spectrophotometrically, and the results were also verified by visual examination. As it is recommended by the CLSI guidelines, for amphotericin B, clotrimazole, econazole, and itraconazole, the MICs were read as the lowest concentrations that prevented any discernible growth (100% inhibition). The MICs of fluconazole and ketoconazole were determined as the lowest concentration of the drug that caused approximately 50% reduction in growth compared to the growth observed in the drug-free medium. While in case of terbinafine, the turbidity allowed corresponded to 80% or more reduction in growth compared to the control well (Clinical and Laboratory Standards Institute, 2008).

### Drug Interaction Tests

Antifungal drug combinations were investigated using the checkerboard microdilution method (Meletiadis et al., 2010) between the commonly applied combinations of antifungals in *A. tamarii* keratitis presented in Table 2, viz., natamycin-econazole, natamycin-itraconazole, and econazole-itraconazole. Antifungal drug dilutions and inocula were prepared based on the CLSI methodology. The final concentration ranges of econazole, itraconazole and natamycin were 2–64, 0.01–0.25, and 0.03–1 μg/ml, respectively. All plates were read spectrophotometrically, and the results were verified by visual examination. Drug interactions were assessed after 48 h incubation based on the fractional inhibitory concentration index (FICI) calculated for all of the wells that corresponded to a MIC. Synergism was defined as FICI ≤ 0.5, 0.5 ≤ FICI ≤ 4 indicated indifference, whereas antagonism was defined as FICI ≥ 4 (Odds, 2003).

**TABLE 2:** Detailed characteristics of the six newly presented cases of keratitis caused by *Aspergillus tamarii* and their comparison to previously reported cases.

**Case no.**	**State, country**	**Age/sex**	**Month of presentation**	**Prior surgery**	**Eye/symptoms**	**Gram- stain/KOH/PAS**	**Injury/risk factor**	**Ulcer severity**	**Hypopyon**	**VA**		**Antifungal therapy**		**Complications**	**Outcome**	**References**
										**Initial**	**Final**	**Topical**	**Systemic**			
Case 1	TN, India	75/F	July	Cataract/IOL	LE/defective vision, pain, redness	+	No	Severe	Yes	LP	NA	NTM, ECN (then CLT), ITC	KTC	Assumed bacterial coinfection	Good response, no follow-up	This study
Case 2	TN, India	60/F	September	No	LE/redness, pain, watering, defective vision	+	No	Severe	No	2/60	2/60	NTM, ECN (then CLT), ITC	KTC	No	Healed, no follow-up	This study
Case 3	K, India	88/M	May	SBCS/IOL	RE/defective vision, pain, redness	−	No	Severe	Yes	LP	NA	NTM, ECN, ITC, AMB	KTC	Bacterial coinfection, vitritis	Evisceration	This study
Case 4	TN, India	75/F	June	No	RE/watering, pain, defective vision	−	Dust	Mild	No	6/24	6/18	NTM, ITC	No	No	Healed	This study
Case 5	TN, India	40/F	June	No	RE/redness, pain and watering	+	Insect	Mild	No	6/12	6/24	NTM, ECN, ITC	KTC	No	Healed	This study
Case 6	TN, India	48/M	June	No	LE/pain, photophobia, defective vision	+	Mud	Severe	Yes	6/24	NA	NTM, ECN, ITC	KTC	No	No response, no follow-up	This study
NA	TN, India	32/F	December	No	LE/pain, redness, defective vision	+	Iron piece	NA	Yes	1/2/60	6/12	NTM, ECN, FLC	KTC	No	Improved, central nebular scar	Kredics et al., 2007
NA	Spain	NA/M	August	LASIK, CRI, vitrectomy, scleral buckling	LE/blurred vision, pain, discharge	+	No injury/contact lens wearer	NA	Yes	6/60	NA	VRC, AMB, NTM	AMB, VRC	No	Improved, extensive corneal scar	Cuadros et al., 2018
Patient 4	Mexico	NA/M	NA	No	RE/NA	+	NA	NA	NA	HM	6/12	VRC	ITC	NA	Improved, no surgery	Al-Hatmi et al., 2019
Patient 16	Mexico	51/M	NA	No	LE/NA	−	NA	NA	NA	HM	LP	VRC after surgery	No	NA	Tectonic KP, vitrectomy	Al-Hatmi et al., 2019
Patient 17	Mexico	51/M	NA	No	LE/NA	+	NA	NA	NA	HM	LP	VRC after surgery	No	NA	Tectonic KP, vitrectomy	Al-Hatmi et al., 2019
Patient 20	Mexico	32/F	NA	No	RE/NA	+	NA	NA	NA	6/60	NA	VRC	No	NA	Tectonic KP, OKP, intraocular lens	Al-Hatmi et al., 2019

*AMB, amphotericin B; CLT, clotrimazole; CRI, corneal ring implantation; ECN, econazole; F, female; HM, hand motion; IOL, intraocular lens implantation; ITC, itraconazole; LE, left eye; LP, light perception; K, Kerala; KP, keratoplasty; KTC, ketoconazole; LASIK, laser *in situ* keratomileusis; M, male; NA, not available; NTM, natamycin; OKP, optical keratoplasty; PAS, Periodic acid-Schiff; RE, right eye; SBCS, simultaneous bilateral cataract surgery; TN, Tamil Nadu; VA, visual acuity; VRC, voriconazole.*

### LC-MS/MS Analysis of CPA

Cyclopiazonic acid was extracted as described before by Chang et al. (2009) with minor modifications. Briefly, the stationary cultures of *A. tamarii* isolates were grown in 2 ml of RPMI-1640 medium (pH 7.0; Sigma-Aldrich) supplemented with 10% heat inactivated fetal bovine serum (FBS; Gibco) at 37°C and simultaneously, in 2 ml of yeast extract sucrose (YES; pH 6.0) medium supplemented with soya peptone at 30°C. After 7 days of incubation, 2 ml of chloroform was added to each sample and vortexed for 30 s. Then, the mixtures were incubated for 2 h at room temperature and vortexed again for 30 s. In order to separate the aqueous and the organic phases, the samples were centrifuged at 9000 × *g* for 15 min. Then, the organic layer was transferred to a clean microcentrifuge tube, air dried and resuspended in 1 ml methanol. Mycelia from each sample were collected and dried in an oven at 70°C for 16 h to determine their dry weight. For the quantitation of CPA, 2 μl of extract was applied onto a Gemini NX C18 50 × 2 mm 3 μ (Phenomenex, United States) column built in a modular Shimadzu Nexera XR HPLC system (Shimadzu, Japan) equipped with a DGU-20A5R degasser, an LC-20AD XR quaternary pump, a SIL-20AXR autosampler and a CTO-10ASVP column thermostat. For the detection, a TSQ Quantum Access (Thermo Fisher Scientific, United States) triple quadrupole mass spectrometer (MS) was connected to the HPLC fitted with an electrospray ionization (ESI) source. The determination was performed according to Moldes-Anaya et al. (2009) with minor modifications. The separation was carried out at 35°C at a flow rate of 0.2 ml/min using gradient elution from 5 mM ammonium acetate buffer/methanol (60/40, v/v) to 100% of methanol in 8 min time, than maintaining this percentage for 5 min and finally decreased to the starting value till pressure stabilization. The detection was in negative ESI, multiple reaction (MRM) mode using the *m/z* 335.1→141.3 (quanty) *m/z* 335.1→181.2 (qualy) transitions, where the collision gas pressure was fixed at 200 MPa and the collision energies (CE) were 30 V for each of the product ions. The MRM method consists of one scan event where the ion injection time was set to 100 ms with a total of 1 μcans/s. The parameters for the ESI interface were adjusted as follows: a spray voltage of 5.5 kV, a capillary temperature of 200°C, a tube lens offset of −167 V, a sheath gas flow rate of 0.6 l/min, and an auxiliary gas flow rate at 6 l/min. The divert valve was applied at 3.5 min and 8.5 min in order to maintain the instrument with a constant performance. The HPLC-MS instrument was controlled with the XCalibur v. 3.1 software (Thermo Fisher Scientific, United States), while the TraceFinder v. 4.1 software (Thermo Fisher Scientific, United States) was applied for data acquisition and evaluation.

### Ethics Statement

PM received an approval from the Institutional Review Board of the Aravind Eye Hospital for the project entitled “PCR based detection of *Fusarium* and *Aspergillus*, and evaluation of their antifungal susceptibilities from patients with keratomycosis attending a tertiary care eye hospital” in 2009. In order to protect the patients’ anonymity, identifying information were not included in the manuscript.

## Results

### Patient Characteristics

The brief summary of clinical data is available for all cases in Table 2. Out of the six isolates, five were isolated from patients residing in towns or cities of Tamil Nadu state, while one from a city of Kerala state. Most patients (*n* = 4) were female, the median age was 67.5. Majority of the infections (*n* = 5) was registered between June and September. The most frequently mentioned symptoms were pain, defective vision and redness. None of the patients had any relevant underlying conditions or reported immunosuppression. Half of the patients mentioned trauma as a predisposing factor, while the other three patients could not recall any injury prior to the infection. The severity of ulcers was recorded as mild in two and severe in four cases. In case of Patients 1, 2, 5, and 6, direct microscopic examination of KOH mounts and Gram-stained smears revealed the presence of fungal elements in the corneal scrapings. Gram-staining and 10% KOH wet mount showed a moderate number of Gram-negative bacilli with no fungal filaments in case of Patient 3, while the corneal scrapings of Patient 4 were negative for both fungal filaments and bacteria. However, cultures were subsequently positive for *Aspergillus* sp. in these latter cases as well.

#### Therapy and Outcome

To sum up, the most common therapeutic approach was the combined topical application of natamycin, itraconazole and econazole eye drops supplemented with systemic ketoconazole treatment. Following this, the ulcer was completely healed in three patients. Evisceration was performed on one patient who developed severe ulcer and did not respond to antifungal therapy. Among the remaining two cases, one patient responded well to the therapy, whereas the other patient showed poor improvement, although both failed to report for the follow-up treatment. The detailed descriptions of each case are as follows:

Patient 1 and 2 were placed on natamycin 5% and econazole 2% eye drops half hourly, itraconazole 1% eye ointment and homatropine 1% eye drops thrice daily, ketoconazole (200 mg), and Dolomed (ibuprofen 600 mg + paracetamol 325 mg) twice daily. Once the culture results (*A. flavus* in both cases) were available, econazole 2% was replaced by clotrimazole 1% eye drops. The second follow-up visit examination of Patient 2 revealed a healing infiltrate. But in case of Patient 1, the corneal infiltrate was not responding to therapy, therefore a coexistent bacterial infection was suspected, and vancomycin 2% eye drops were added. Over the next 3 days the infiltrate resolved well and at the time of discharge 2 weeks after admission there was only a minimal deep stromal infiltrate.

Based on the results of the direct examinations of the scrapings, Patient 3 was started on ciprofloxacin 0.3% and tobramycin 0.3% eye drops six times a day along with homatropine 1% eye drops, oral ciprofloxacin (500 mg) twice a day and intravenous garamycin (80 mg) twice a day. However, the subsequent growth of fungal colonies on culture media was identified as *A. flavus*. The next day antibiotics were tapered to four times a day. Then, natamycin 5% and econazole 2% were added on an hourly basis along with itraconazole 1% eye ointment and homatropine 1% eye drops thrice daily, ketoconazole and Dolomed twice daily. Amphotericin B (50 μg/ml) half-hourly was added on the third day. However, by the fifth day the infection had progressed to involve the sclera and the patient complained of radiating pain to the scalp. A repeated ultrasonography showed reactionary vitritis with mild thickening of the retina*-*choroid*-*sclera complex. As there was no response to therapy and the visual prognosis was very poor, evisceration was offered to the patient and was done 5 days after presentation to the hospital.

Patient 4 was placed on natamycin 5% and moxifloxacin 0.5% eye drops hourly, along with itraconazole 1% eye ointment and homatropine 1% eye drops thrice daily, and Dolomed twice daily. Three days later, the infiltrate had resolved completely.

Patient 5 and 6 were placed on 5% natamycin and econazole 2% eye drops half hourly, itraconazole 1% eye ointment and homatropine 1% eye drops thrice daily; oral ketoconazole (200 mg) and Dolomed twice daily. Patient 5 showed good response to therapy while in case of Patient 6 the infiltrate continued to be active.

### Molecular Identification

**FIGURE 1:**
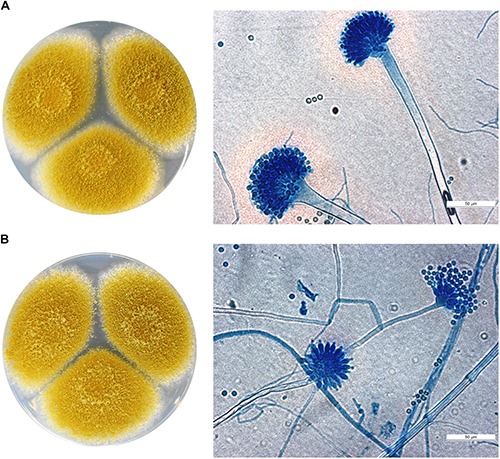
Macro- and micromorphology (after lactophenol cotton blue staining) of *A. tamarii* **(A)** SZMC 2439 and **(B)** SZMC 2393 grown on potato dextrose agar plates for 5 days at 37°C. Scale bar: 50 μm.

The six isolates of the study were initially identified as members of the genus *Aspergillus*, i.e., *Aspergillus* sp. (*n* = 1), *A. flavus* (*n* = 4), and *A. fumigatus* (*n* = 1) based on morphological examinations (Figure 1 and Table 1). Molecular identification did not confirm these preliminary data, since both the ITS and the calmodulin sequences of the six isolates showed ≥99% similarity to sequences of the CBS 104.13 type strain of *A. tamarii*.

### Antifungal Drug Susceptibilities and Drug Interactions

The obtained MIC values are summarized in Table 3. It could be noted that most of the MICs of fluconazole and natamycin were ≥64 μg/ml. Amphotericin B, clotrimazole, econazole, itraconazole, ketoconazole and terbinafine exerted good antifungal activity (MIC ≤ 2 μg/ml) against all the six isolates. Among them, terbinafine proved to be the most effective drug *in vitro* with a MIC of 0.008 μg/ml.

**TABLE 3:** Minimum inhibitory concentration (MIC; μg/ml) values of antifungal agents for six *Aspergillus tamarii* isolates from human keratomycosis.

**Isolate**		**SZMC 2393**	**SZMC 2404**	**SZMC 2428**	**SZMC 2434**	**SZMC 2438**	**SZMC 2439**	**MIC range (μg/ml)**	**GM MIC (μg/ml)**
Antifungal agent	AMB	1	2	2	1	0.5	2	0.5–2	1.3
	CLT	0.25	0.5	1	1	0.25	0.25	0.25–1	0.4
	ECN	0.5	0.25	1	0.25	0.25	0.25	0.25–1	0.4
	FLC	>64	>64	>64	>64	>64	>64	>64	>64
	ITC	0.25	0.06	0.125	0.06	0.06	0.125	0.06–0.25	0.1
	KTC	2	2	1	1	1	2	1–2	1.4
	NTM	≥64	≥64	≥64	16	≥64	≥64	16 – ≥64	≥64
	TRB	0.008	0.008	0.008	0.008	0.008	0.008	0.008	0.008

*AMB, amphotericin B; CLT, clotrimazole; ECN, econazole; FLC, fluconazole; GM, geometric mean; ITC, itraconazole; KTC, ketoconazole; NTM, natamycin; SZMC, Szeged Microbiological Collection, University of Szeged, Szeged, Hungary; TRB, terbinafine.*

Most interactions between the tested antifungal drug combinations were indifferent (Table 4). For the combination of natamycin and econazole the FICIs ranged from 0.49 to 1.03, synergism was detected against one isolate (i.e., SZMC 2434). For econazole and itraconazole the FICIs were between 0.58 and 0.98, indicating no interactions between the two drugs. Synergism was detected between natamycin and itraconazole against *A. tamarii* SZMC 2428 and SZMC 2438 isolates (FICI = 0.49), while against the rest of the isolates, the FICIs were between 0.54 and 0.75 suggesting indifferent interactions.

**TABLE 4:** *In vitro* combinations of antifungal drugs against six *Aspergillus tamarii* isolates from human keratomycosis.

**Isolate**		**FICI_min_/interpretation^∗^**					
		**SZMC 2393**	**SZMC 2404**	**SZMC 2428**	**SZMC 2434**	**SZMC 2438**	**SZMC 2439**
Antifungal agent	ECN-ITC	0.72/I	0.58/I	0.58/I	0.58/I	0.98/I	0.98/I
	ECN-NTM	1.03/I	0.61/I	1.03/I	0.49/S	0.54/I	0.98/I
	ITC-NTM	0.66/I	0.75/I	0.49/S	0.73/I	0.49/S	0.54/I

*ECN, econazole; FICI_*min*_, minimal fractional inhibitory concentration index; ITC, itraconazole; NTM, natamycin; SZMC, Szeged Microbiological Collection, University of Szeged, Szeged, Hungary. ^∗^I, indifference (0.5 < FICI ≤ 4); S, synergism (FICI ≤ 0.5).*

### CPA-Producing Abilities

**FIGURE 2:**
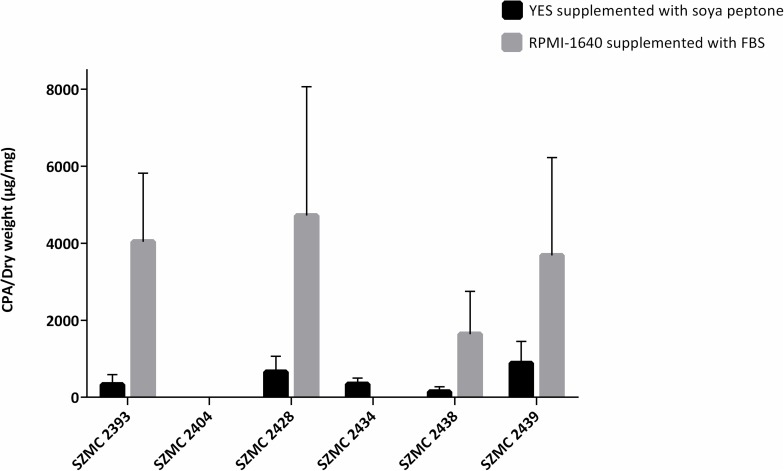
Cyclopiazonic acid (CPA) producing ability (μg/mg mycelial dry weight) of the stationary cultures of *A. tamarii* isolates grown in yeast extract sucrose (YES) medium supplemented with soya peptone and in RPMI-1640 supplemented with fetal bovine serum (FBS) for 7 days in the dark. Results are the mean of two independent experiments with two individual replicates. The limit of quantitation was 500 ng/ml.

The CPA-producing abilities of the six *A. tamarii* isolates were investigated in two different media, viz., YES supplemented with soya peptone and in RPMI-1640 medium supplemented with FBS. As shown in Figure 2, four of the six isolates were capable to produce CPA in RPMI-1640 medium, while five isolates proved to be toxinogenic in YES. Although *A. tamarii* isolates grew poorly in RPMI-1640 medium (mean mycelial dry weight: 3.1 ± 1.0 mg), they produced a relatively high amount of CPA, ranging between 3.2 ± 1.0 μg/ml and 7.1 ± 4.7 μg/ml (mean CPA content: 6.5 ± 2.4 μg/ml). This is comparable to the CPA content of the YES samples, where CPA concentration ranged from 3.8 ± 4.1 μg/ml to 22.2 ± 16.7 μg/ml (mean CPA content: 12.5 ± 7.5 μg/ml). However, compared to RPMI-1640, this CPA concentration was attributed to a biomass higher than one order of magnitude in YES (mean mycelial dry weight: 51.7 ± 5.2 mg).

## Discussion

The currently presented six cases and the previous reports of *A. tamarii* keratitis cases suggest that this species is a rare but regularly emerging pathogen of ocular infections. Although it was mainly reported from India before (Kredics et al., 2007; Ghosh et al., 2016; Manikandan et al., 2019), its occurrence is not restricted to this country: a contact lens associated case from Spain, and four cases from Mexico have just been described recently (Cuadros et al., 2018; Al-Hatmi et al., 2019). Seasonal variations are usually reported for keratitis. In a recent study from South India, most of the fungal isolates (80%) were obtained during the months of July–September (Ranjini and Waddepally, 2016). Similarly, Bharathi et al. (2003) isolated the highest number of fungal strains between June and September. In accordance with these reports, five of the six *A. tamarii* isolates in this study were isolated within this 4- months period of the year. Environmental factors (e.g., humidity, temperature, wind, etc.), which are also responsible for these seasonal variations, may affect the geographical distribution of the infecting agents as well (Leck et al., 2002; Thomas and Kaliamurthy, 2013). Although aspergilli are more frequently reported from keratitis in North India, it is not the case with *A. tamarii*. This species is found mostly in tropical regions (Aries et al., 2018), and were reported more commonly from South India (*n* = 7; Kredics et al., 2007; This study) than from the Northern part of the country (Ghosh et al., 2016).

Trauma was a key predisposing factor for the infection, as it was expected based on previous reports (Kredics et al., 2007; Thomas, 2009; Manikandan et al., 2013). Additionally, Manikandan et al. (2013) listed co-existing ocular conditions and systemic diseases, such as diabetes mellitus and hypertension, among the risk factors for *Aspergillus* keratitis (Manikandan et al., 2013). Contact-lens associated cases of fungal keratitis are mainly reported from developed countries (Ansari et al., 2013). Accordingly, the single case of *A. tamarii* keratitis in a contact lens wearer was reported from Spain (Cuadros et al., 2018).

Our results presented in Table 1 underline the importance of using molecular identification methods in order to avoid misdiagnosis. Out of the six isolates in this study, five were identified at the species level based on morphological characteristics: four as *A. flavus*, and one as *A. fumigatus*, but none of them as *A. tamarii* (Table 1). In line with our experiences, Tam et al. (2014) suggested that β-tubulin or the calmodulin genes are the best choice for identifying *A. flavus*, *A. nomius*, and *A. tamarii*. According to the authors, matrix-assisted laser-desorption/ionization time-of-flight mass spectrometry (MALDI-TOF MS) might also be used for an accurate identification, however, this method requires a comprehensive database including different strains of a wide range of species. Similarly, the recent work of Cuadros et al. (2018) concludes that MS combined with phenotypic analyses is a useful tool for the diagnosis of *A. tamarii* and other rare fungal pathogens. Recently, Al-Hatmi et al. (2019) also emphasized the importance of using molecular identification methods for the diagnosis of *A. tamarii* and other rare fungal pathogens. In this way, it is possible that studies using morphological identification methods alone for the diagnosis of *A. tamarii*, may not be completely reliable (Degos et al., 1970; Paludetti et al., 1992; Kamble, 2015; Aries et al., 2018).

Antifungal susceptibility results of the present study are comparable to the reports listed in Table 5. Natamycin had no inhibitory effect on the *in vitro* growth of *A. tamarii* isolates of the study, which is similar to the results of Kredics et al. (2007) and Manikandan et al. (2019) obtained for Indian isolates, but it is in contrast to the study of Al-Hatmi et al. (2019) who reported a lower MIC range of 4–16 μg/ml against four Mexican isolates. Despite the conflicting *in vitro* data, the vast majority of patients diagnosed with fungal keratitis is usually treated with natamycin eye drops alone or in combination with other topical antifungal drugs (e.g., itraconazole) (Ghosh et al., 2016) and it proved to have favorable effects in patients with *Aspergillus* keratitis previously (Kalavathy et al., 2005). Natamycin was administered to eight patients diagnosed with *A. tamarii* keratitis as well (Table 2), raising the possibility that in combination with other antifungal agents (i.e., econazole and itraconazole) it could facilitate the reduction of the severity of infections. However, our *in vitro* observations could not support this theory, since the predominance of indifferent interactions was revealed between natamycin and econazole and between natamycin and itraconazole as well. Whereas, synergism was only detected between the combinations of natamycin-itraconazole and natamycin-econazole against three *A. tamarii* isolates.

**TABLE 5:** Minimum inhibitory concentration (MIC; μg/ml) values of antifungal agents for the *Aspergillus tamarii* isolates reported previously from various human infections.

**References**		**Kredics et al., 2007*n* = 1 (*E*-test)**	**Baranyi et al., 2013*n* = 4 (*E*-test)**	**Cuadros et al., 2018*n* = 1 (E-test)**	**Manikandan et al., 2019*n* = 1 (BMD)**	**Al-Hatmi et al., 2019*n* = 4 (BMD)**	**Castro et al., 2019**	
							***n* = 3 (BMD)**	***n* = 3 (*E*-test)**
Antifungal agent	AMB	0.125	0.002–0.008	0.25	≤0.5	0.5–1 (0.59)	0.12	0.5–2
	AND	–	0.38–1	–	–	–	0.015–0.03	–
	CLT	–	–	–	≤0.5	–	–	–
	CSP	–	0.016–0.25	0.5	–	–	≤0.03	–
	ECN	0.064	–	–	≥1	–	–	–
	FLC	>256	>32	>256	–	–	–	–
	ITC	0.064	0.38–0.75	–	≤0.25	0.5–2 (0.84)	≤ 0.03–0.06	0.5–1
	KTC	0.25	–	–	≥1	–	–	–
	MCF	–	–	–	–	–	≤0.03	0.06–0.25
	NTM	>1024	–	–	≥32	4–8(5.65)	–	–
	PSC	–	0.125–0.94	0.094	–	–	≤ 0.03–0.06	0.125–0.25
	TRB	–	–	–	–	–	–	–
	VRC	0.125	0.125–0.19	–	≥1	0.5–4 (0.133)	0.06–0.25	0.64–0.125

*We only included data from papers using molecular or MALDI-TOF MS analyses for the confirmation of morphological identification. AMB, amphotericin B; AND, anidulafungin; BMD, broth microdilution; CLT, clotrimazole; CSP, caspofungin; ECN, econazole; FLC, fluconazole; ITC, itraconazole; KTC, ketoconazole; MCF, micafungin; NTM, natamycin; PSC, posaconazole; TRB, terbinafine; VRC, voriconazole.*

Just as was the case with natamycin, fluconazole was not able to inhibit the *in vitro* growth of *A. tamarii* isolates in the current investigation (Table 3). However, this antifungal agent lessened the severity of *Aspergillus* keratitis in a rabbit model (Avunduk et al., 2003), the favorable outcome of fluconazole therapy in *Aspergillus* keratitis has not been confirmed in humans yet.

As it is shown in Table 5, beside the antifungal agents examined in this study, anidulafungin, caspofungin, micafungin, posaconazole and voriconazole also proved to be active against *A. tamarii in vitro* (Kredics et al., 2007; Baranyi et al., 2013; Al-Hatmi et al., 2019; Castro et al., 2019; Manikandan et al., 2019). Moreover, voriconazole had the best *in vitro* activity against *A. tamarii* isolates in the study of Al-Hatmi et al. (2019), and it was also used to treat successfully one out of the four patients diagnosed with *A. tamarii* keratitis without any surgery. In the present study, terbinafine was the most active antifungal agent against all the six isolates. Its *in vitro* efficiency against *A. tamarii* has not been investigated yet (Table 5), but the topical application of 0.25% terbinafine eye drops on fungal – mainly *Aspergillus* and *Fusarium* – keratitis has been compared to 5% natamycin in a retrospective clinical study before (Liang et al., 2009). Although the majority of patients showed favorable response to terbinafine therapy, they needed a longer treatment course compared to the patients in the natamycin-group. Nonetheless, terbinafine eye drops might be a good alternative in the future for the treatment of fungal keratitis, especially in countries where natamycin or voriconazole is not available.

Our tests revealed, that the antifungal drug susceptibility profile of *A. tamarii* is similar to those of the other keratitis-derived members of section *Flavi* (Baranyi et al., 2013). In the present study, only the MICs of amphotericin B (MIC range: 0.5–2 μg/ml) differed significantly from the previously published values for *A. flavus* (MIC range: 2–12 μg/ml; Baranyi et al., 2013). Although the CLSI epidemiological cut-off (ECOFF) values are not available yet for *A. tamarii*, the ECOFF of amphotericin B for other aspergilli (2 μg/ml for *A. fumigatus*, *A. flavus*, *A. niger*; 4 μg/ml for *A. terreus*) and the aforementioned MIC range obtained for *A. tamarii* isolates suggest, that the members of this species may respond well to an amphotericin B therapy (Lass-Flörl, 2014).

Treatment options seem to vary between countries (Table 2; Kredics et al., 2007; Cuadros et al., 2018; Al-Hatmi et al., 2019). In India, the combination of topical natamycin, econazole and itraconazole supplemented with systemic ketoconazole was the most common treatment of choice (this study; Kredics et al., 2007). In Spain, the patient’s antifungal therapy started with topical 1% voriconazole and systemic liposomal amphotericin B, and after he was discharged, it was changed to topical 5% natamycin and 1% voriconazole, and oral voriconazole (200 mg/12 h) (Cuadros et al., 2018). In Mexico, 1% voriconazole was the topical drug of choice (Al-Hatmi et al., 2019), while systemic therapy was applied in case of one patient who received oral itraconazole (200 mg/24 h). Improvement was detected in one patient without surgery, while it was necessary in the other three patients (Al-Hatmi et al., 2019). The patient’s condition in Spain was also improved (Cuadros et al., 2018). Whereas in India, five patients showed improvement but two showed no response to therapy despite the similar combinations of antifungal drugs applied in all the seven cases (this study; Kredics et al., 2007).

Cyclopiazonic acid serves as a key plant pathogenicity factor for the closely related species *A. flavus* (Chalivendra et al., 2017), and it may also influence the virulence of aspergilli in animal and human hosts by contributing to the deterioration of the patient’s condition through its neurotoxic and immunomodulatory effects (Keblys et al., 2004; Chalivendra et al., 2017). Production of CPA by *A. tamarii* was firstly reported by Dorner et al. (1983), where, 96% (22/23) of the investigated isolates were able to produce this mycotoxin. Similarly, the current study confirmed the CPA-producing ability for five out of the six examined keratitis isolates. In contrast to the present study, Varga et al. (2011) observed that *A. tamarii* strains produced a lower amount (1.74–2.49 ppm) of CPA in modified Czapek Dox medium. Whereas, in comparison to *A. flavus* isolates (32.5 ± 2.5 – 57.9 ± 4.1 μg/ml in YES with soytone) (Chang et al., 2009), the currently presented six *A. tamarii* isolates possessed a lower CPA-productivity in modified YES and RPMI-1640 media as well. Comparable results of CPA production by other clinical *A. tamarii* isolates are not available in the literature.

In conclusion, our results confirm that *A. tamarii* is an emerging human pathogenic species associated with keratomycoses in India. The number of studies on *A. tamarii* infections, where both molecular identification and antifungal susceptibility testing were performed and clinical data are also available, is very small (Kredics et al., 2007; Al-Hatmi et al., 2019; this study). However, these researches are crucial to answer the question whether the use of molecular identification approaches in clinical practice could improve the outcome of fungal eye infections. *In vitro* antifungal susceptibility testing revealed that terbinafine might be a good alternative as a topical eye drop or ointment to treat *A. tamarii* keratitis in the future. In addition, we also revealed that drug interactions between the most commonly applied antifungal agents were mainly indifferent, while synergistic interactions were registered in three cases. Although *A. tamarii* seems to respond well to antifungal agents and their combinations *in vitro*, poor outcomes have been registered as well among the six presented cases. This fact indicates, that not just the treatment of choice, but the unique characteristics of the patient and the infecting strain (e.g., its mycotoxin producing ability) might also affect the final outcome of a therapy. Our observations on the ability of *A. tamarii* strains to produce a high amount of CPA despite their poor growth in RPMI-1640 will probably facilitate further studies to examine whether this mycotoxin is able to promote tissue damage and exacerbate the patient’s condition during an eye infection.

## Data Availability Statement

Nucleotide sequences generated for this study can be found in the NCBI GenBank, MG907190, MG912921, MG907191, MG912922, MG907192, MG912923, MG907193, MG912924, MG907194, MG912925, MG907195, and MG912926.

## Author Contributions

PM, TP, and MH contributed to the design and implementation of the research and participated in drafting the manuscript. PM, RR, VN, and CS collected the clinical data. SK and NK participated in the molecular identification and the mycotoxin extraction. AS performed the LC-MS/MS analysis. MH performed the antifungal susceptibility tests, designed the tables and the figures, and drafted the manuscript. CV, BA, AB, and LK contributed to the evaluation of the results and helped in drafting the manuscript. All authors reviewed and approved the final version of the manuscript.

## Conflict of Interest

The authors declare that the research was conducted in the absence of any commercial or financial relationships that could be construed as a potential conflict of interest.

## Footnotes

1 http://www.ncbi.nlm.nih.gov/blast/

2 http://www.wfcc.info/ccinfo/collection/by_id/987
